# Safety and pharmacokinetics of teplizumab in children less than 8 years of age with stage 2 type 1 diabetes

**DOI:** 10.1007/s00125-025-06586-1

**Published:** 2025-11-06

**Authors:** Stephen E. Gitelman, Kimber Simmons, Jennifer L. Sherr, Steven B. Leichter, Teresa Quattrin, William E. Russell, Bhuvana Sunil, Steven M. Willi, Laura A. Knecht, Elisabeth Niemoeller, Idlir Licaj, Wolfgang Schmider, Diana Miller, Linda A. DiMeglio

**Affiliations:** 1 Department of Pediatrics, University of California at San Francisco, San Francisco, CA USA; 2 Diabetes Center, University of California at San Francisco, San Francisco, CA USA; 3 Barbara Davis Center for Diabetes/University of Colorado School of Medicine, Aurora, CO USA; 4 Department of Pediatrics, Pediatric Endocrinology, Yale University School of Medicine, New Haven, CT USA; 5 Piedmont Endocrinology Columbus, Columbus, GA USA; 6 Department of Internal Medicine, Mercer University School of Medicine, Columbus, GA USA; 7 Department of Pediatrics, Jacobs School of Medicine and Biomedical Sciences, University at Buffalo, Buffalo, NY USA; 8 Department of Pediatrics, Vanderbilt University Medical Center, Nashville, TN USA; 9 Department of Cell and Developmental Biology, Vanderbilt University School of Medicine, Nashville, TN USA; 10 Mary Bridge Children’s Hospital, Tacoma, WA USA; 11 Division of Endocrinology and Diabetes, The Children’s Hospital of Philadelphia, Philadelphia, PA USA; 12 Department of Pediatrics, Perelman School of Medicine, University of Pennsylvania, Philadelphia, PA USA; 13 Sanofi, Morristown, NJ USA; 14 Sanofi, Frankfurt, Germany; 15 Sanofi, Paris, France; 16 Cytel, Madrid, Spain; 17 Division of Pediatric Endocrinology and Diabetology, Department of Pediatrics, Indiana University School of Medicine, Indianapolis, IN USA

**Keywords:** Child, Disease progression, Immunogenicity, Paediatrics, Pharmacodynamics, Pharmacokinetics, Pharmacology, Safety, Teplizumab, Type 1 diabetes

## Abstract

**Aims/hypothesis:**

Teplizumab is approved in the USA and seven other countries to delay stage 3 type 1 diabetes onset in individuals ≥8 years of age with stage 2 type 1 diabetes. As part of a US Food and Drug Administration post-marketing requirement, this study evaluated the safety, tolerability and pharmacokinetics of teplizumab in children aged <8 years with stage 2 type 1 diabetes.

**Methods:**

The PETITE-T1D trial is a 2 year single-arm, open-label, multicentre study of 23 children <8 years of age with stage 2 type 1 diabetes. Participants received a 14 day teplizumab course. A prespecified interim analysis was performed after 15 participants completed 1 year of follow-up and included all 23 participants. Primary endpoints included treatment-emergent adverse events (TEAEs), TEAEs causing treatment discontinuation, and serious adverse events (SAEs). Other endpoints assessed immunogenicity, pharmacokinetics, pharmacodynamics and time from study treatment to stage 3 type 1 diabetes.

**Results:**

Mean participant age was 4.8 years (range 1.7–6.8). Median follow-up duration was 51.9 weeks (range 3.9–77.1). All participants experienced one or more TEAE, with most being mild to moderate. No grade 4 or 5 TEAEs were reported. Three participants (13%) had TEAEs leading to teplizumab discontinuation: anaemia, elevated liver enzymes and maculo-papular rash. Two participants (9%) each had two SAEs. Serum teplizumab concentrations peaked at day 14. Two participants progressed to stage 3 type 1 diabetes. The estimated probability of lack of progression to stage 3 was 89.6% (95% CI 64.3%, 97.3%) at the time of interim analysis.

**Conclusions/interpretation:**

Teplizumab was safe and well tolerated in children <8 years of age with stage 2 type 1 diabetes. Adverse events were consistent with those seen in previous studies, with no new safety risks identified. Two participants progressed to stage 3 type 1 diabetes during the observation period; surveillance is ongoing.

**Trial registration:**

ClinicalTrials.gov NCT05757713.

**Graphical Abstract:**

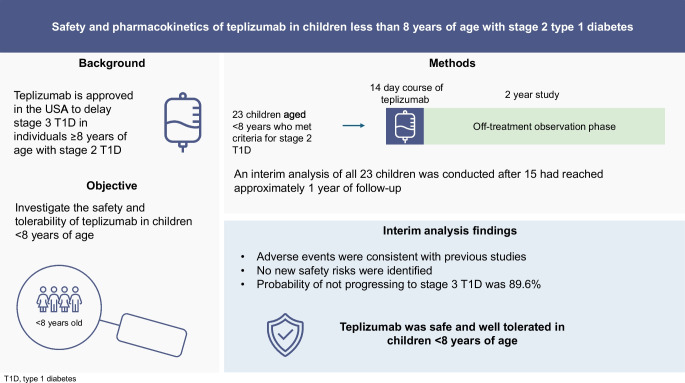

**Supplementary Information:**

The online version contains peer-reviewed but unedited supplementary material available at 10.1007/s00125-025-06586-1.


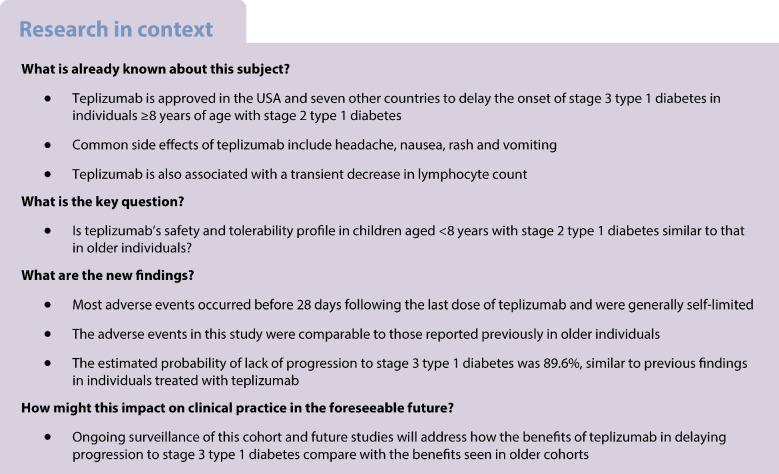


## Introduction

Type 1 diabetes is one of the most common childhood diseases and its incidence is increasing in children [1–3]. Although the T cell-dependent autoimmune destruction of beta cells may be clinically silent in early-stage disease, the presence of two or more islet autoantibodies and dysglycaemia [4], referred to as stage 2 type 1 diabetes, is associated with a high likelihood of progression to stage 3 [5, 6]. Notably, the progression to stage 3 is most rapid in young children [7].

Managing the disease in young children is challenging and places a high burden on caregivers [8, 9]. Studies have shown that only approximately 20% of children on insulin therapy reach the target HbA_1c_ of <53 mmol/mol (<7%) [10–12]. Moreover, young children often exhibit selective eating, variable levels of physical activity and difficulty expressing symptoms and thoughts, which places them at higher risk for hypoglycaemia [8, 13]. Delaying stage 3 onset with disease-modifying therapy may postpone the need for insulin therapy to a time when children have greater physical and developmental maturity, thus permitting time for their brains to become more mature and less susceptible to the negative effects of disordered glycaemic regulation [14–20].

In 2022, the US Food and Drug Administration (FDA) approved teplizumab, a humanised immunoglobulin G1 antibody that binds the T cell receptor CD3ε chain, to delay onset of stage 3 type 1 diabetes in individuals aged ≥8 years with stage 2 type 1 diabetes [21]. The approved 14 day course of i.v. teplizumab infusion leads to partial T cell exhaustion and reduced expansion of autoreactive CD8^+^ T cells [22]. Data have shown that teplizumab treatment in individuals aged ≥8 years with stage 2 type 1 diabetes preserves beta cell function and results in a median delay of progression to stage 3 of 2‒3 years, although not all treated individuals respond [22, 23]. A consistent finding, related to teplizumab’s mechanism of action, has been a transient decrease in lymphocyte count with treatment [24, 25]. Importantly, rates of infections with teplizumab have been similar to those in control individuals [24]. The most common side effects associated with teplizumab include headache, nausea, rash and vomiting [25]. Less common adverse events include cytokine release syndrome (CRS) and elevated liver function tests [24]. Of the CRS cases reported, most were mild to moderate and treated with over-the-counter antipyretics, antihistamines and/or antiemetics [21, 24].

Despite the difficulty in managing stage 3 type 1 diabetes in young children, teplizumab has not been previously investigated in children aged <8 years and is not currently approved for this population [7, 25–31]. Subgroup analyses have suggested that teplizumab is similarly efficacious in children aged ≥8 years and adults [31], supporting the investigation of teplizumab in younger children. The primary objective of the PETITE-T1D study was to investigate the safety and tolerability of teplizumab in children aged <8 years with stage 2 type 1 diabetes. Additionally, the immunogenicity, pharmacokinetics, pharmacodynamics and elapsed time to progression to stage 3 type 1 diabetes were evaluated.

## Methods

### Study design

PETITE-T1D (ClinicalTrials.gov NCT05757713) is a 2 year single-arm, open-label, multicentre study to assess the safety and pharmacokinetics of teplizumab in children aged <8 years with stage 2 type 1 diabetes. The study took place at nine centres in the USA and was initiated on 25 July 2023 (first participant enrolled). The study was conducted as a US FDA post-marketing requirement for the approved indication [21]. This study was approved by an institutional review board/independent ethics committee and was conducted in accordance with the principles of the Declaration of Helsinki. Participants or their legal representatives provided informed written consent before enrolment in the study. A data monitoring committee reviewed safety data on an ongoing basis. This report provides the results of a prespecified interim analysis with a cut-off date of 18 February 2025 that was conducted after 15 participants had reached approximately 1 year of post-treatment follow-up. Data from all enrolled participants (*N*=23) are included in the analysis. The sample size was chosen to evaluate safety and tolerability objectives.

### Participants

Participants were aged <8 years with stage 2 type 1 diabetes. Stage 2 type 1 diabetes was defined as positivity for two or more islet autoantibodies (i.e. GADA, IA-2A, IAA, ZnT8A or ICA) and presence of dysglycaemia without overt hyperglycaemia (i.e. fasting plasma glucose 5.6–6.9 mmol/l [100–125 mg/dl], 2 h plasma glucose 7.8–11.0 mmol/l [140–199 mg/dl], HbA_1c_ 39–47 mmol/mol [5.7–6.4%] or ≥10% increase in HbA_1c_), in accordance with the ADA Standards of Care [32, 33]. Individuals with clinically significant alterations in haematological parameters, liver function test abnormalities, active infections, recent or planned live vaccinations (within 8 weeks before day 1 and within 12 months after completing dosing) or recent or planned inactivated (killed) virus vaccinations (within 2 weeks before day 1 and continuing until 6 weeks after completing dosing) were excluded.

### Administration of teplizumab and additional therapies

**Fig. 1:**
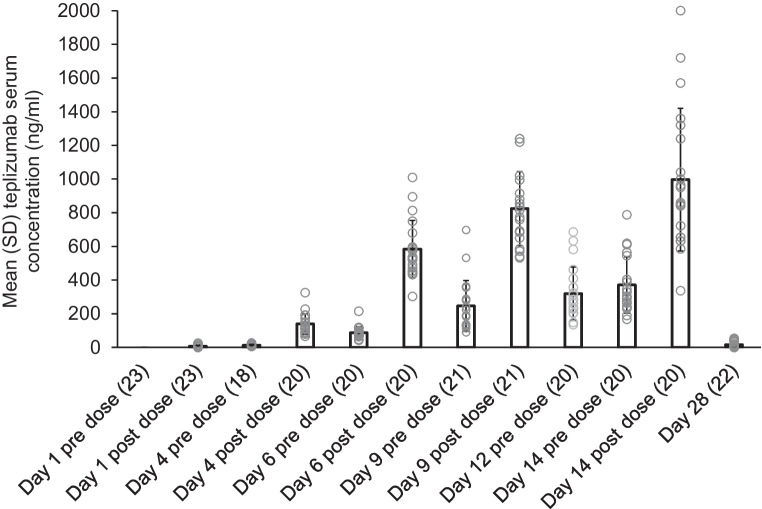
Teplizumab serum concentrations (pharmacokinetics population). The pharmacokinetics population was defined as all enrolled participants who provided one or more post-baseline pharmacokinetics sample. The number of participants analysed at each time point is indicated in parentheses. Unfilled circles show individual participant data

Teplizumab was administered by i.v. infusion, via peripheral catheter, midline catheter or peripherally inserted central catheter (PICC), once daily from day 1 to day 14. The dose of teplizumab administered was the approved dose for individuals with stage 2 type 1 diabetes aged ≥8 years, derived per m^2^ (electronic supplementary material [ESM] Fig. 1) [21]. Participants received oral premedication on days 1–5 consisting of a non-steroidal anti-inflammatory drug or acetaminophen and an antihistamine, plus an antiemetic as needed. On subsequent dosing days, premedication was administered per investigator’s judgement.

### Assessments

The primary endpoints of the trial were treatment-emergent adverse events (TEAEs), adverse events of special interest (AESIs), TEAEs leading to withdrawal, serious adverse events, clinical laboratory parameters and vital signs of clinical importance. Prespecified AESIs in this study are described in ESM Methods. A TEAE was defined as an adverse event that occurred after administration of the first dose of the study drug up to the end of the study. The severity of TEAEs was graded according to National Cancer Institute’s Common Terminology Criteria for Adverse Events (CTCAE) version 5.0 (https://dctd.cancer.gov/research/ctep-trials/for-sites/adverse-events). The secondary endpoints and time points included for the interim analysis were serum concentrations of teplizumab pre and post infusion, antidrug antibody titres and presence of neutralising antibodies (NAbs), and CD3 receptor occupancy by teplizumab (timing of assessments is provided in ESM Methods). Exploratory endpoints included in the interim analysis were time from study treatment to development of stage 3 type 1 diabetes and HbA_1c_ level. Samples were analysed at a central laboratory, except for haematology and clinical chemistry parameters during the 14 day treatment course. Diagnosis of stage 3 type 1 diabetes was based on ADA Standards of Medical Care 2022 and 2025 criteria [32, 33].

### Statistical analyses

This is a prespecified interim analysis with a cut-off date of 18 February 2025, conducted after 15 participants had reached approximately 1 year of post-treatment follow-up (week 52 assessment visit). Data are presented in a descriptive manner, with no formal statistical inferences. The 1 year event-free probability estimate for progression to stage 3 type 1 diabetes was obtained from Kaplan‒Meier survival estimates using the Greenwood formula. Safety data are reported for the enrolled population, defined as all participants who received at least one dose of teplizumab. The immunogenicity, pharmacokinetics and pharmacodynamics populations were defined as all enrolled participants who provided at least one post-baseline antidrug antibody, pharmacokinetics or pharmacodynamics sample, respectively. This was a small study (*n*=23) with primary objectives of safety and tolerability of teplizumab; the protocol did not plan for formal statistical inference or powered subgroup comparisons by sex or gender, nor an assessment of sex or gender differences. Nevertheless, baseline age and sex distribution and descriptive safety summaries (counts and percentages) are presented. Sex was determined by self-report.

## Results

### Participants

Table 1 provides the demographic and disease characteristics of the 23 participants at baseline. Thirty-two individuals were screened for enrolment in the study and nine were deemed ineligible (eight did not meet the criteria for stage 2 type 1 diabetes diagnosis and one had an asymptomatic Epstein‒Barr virus infection) (ESM Fig. 2). The mean age of enrolled participants was 4.8 years (minimum 1.7 years, maximum 6.8 years), 52.2% were female and 95.7% were White. The mean HbA_1c_ at baseline was 37 mmol/mol (SD 5.5) (5.5% [SD 0.5]).

**Table 1:** Demographic and disease characteristics at baseline (enrolled population)

Characteristic	Teplizumab (*N*=23)^a^
Age at day 1, years	
Mean (SD)	4.8 (1.2)
Median	4.9
Min., max.	1.7, 6.8
Age group at day 1	
<2 years	1 (4.3)
2 to <5 years	12 (52.2)
5 to <8 years	10 (43.5)
Sex	
Female	12 (52.2)
Male	11 (47.8)
Race^b^	
White	22 (95.7)
Black or African American	0
Asian	1 (4.3)
American Indian or Alaskan Native	0
Native Hawaiian or Other Pacific Islander	0
Other	0
Unknown	0
Ethnicity^b^	
Hispanic or Latino	3 (13.0)
Not Hispanic or Latino	19 (82.6)
Unknown	1 (4.3)
BSA at baseline, m^2^	
Mean (SD)	0.74 (0.11)
Median	0.73
HbA_1c_ at baseline, mmol/mol^c^	
Mean (SD)	37 (5.5)
Median	37
Min., max.	29, 48
HbA_1c_ at baseline, %^c^	
Mean (SD)	5.5 (0.5)
Median	5.5
Min., max.	4.8, 6.5
Type of T1D-related autoantibody, *n*/*m* (%)^d^	
ICA	11/13 (84.6)
GADA	19/23 (82.6)
IAA	20/23 (87.0)
IA-2A	15/22 (68.2)
ZnT8A	17/23 (73.9)
No. of positive autoantibodies	
2	3 (13.0)
3	8 (34.8)
4	8 (34.8)
5	4 (17.4)
Method for confirmation of dysglycaemia without overt hyperglycaemia	
HbA_1c_	12 (52.2)
2 h plasma glucose	4 (17.4)
Fasting plasma glucose	3 (13.0)
OGTT	7 (30.4)
Relatives with a history of T1D^e^	
Yes	20 (87.0)
No	3 (13.0)
Relation	
Parent	11 (55.0)
Sibling	8 (40.0)
Grandparent	5 (25.0)
Uncle	3 (15.0)
Aunt	2 (10.0)
Cousin	2 (10.0)

Data are *n* (%) unless stated otherwise

The enrolled population was defined as all participants who received one or more doses of teplizumab. The baseline value was defined as the most recent value collected prior to administration of the first dose of teplizumab. All participants had a prior screening visit to determine eligibility

^a^Data were available for all 23 participants unless otherwise specified

^b^Race and ethnicity were self-reported by participants and caregivers

^c^The baseline HbA_1c_ value for one participant (48 mmol/mol [6.5%]) was taken from the first day of study drug treatment (day 1 assessment); the participant’s screening value (prior to day 1, which was used to determine study eligibility) was 46 mmol/mol (6.4%)

^d^For a given autoantibody, ‘*n*’ is the number of positive tests and ‘*m*’ is the number of participants tested

^e^Individuals without relatives with type 1 diabetes either were self-identified by the individuals or caregivers or were identified by the investigators for potential enrolment in the study, in the same way as individuals with relatives with type 1 diabetes

BSA, body surface area; T1D, type 1 diabetes

### Treatment course

At the cut-off date for the interim analysis, the median follow-up duration was 51.9 weeks, with a minimum of 3.9 weeks and a maximum of 77.1 weeks. All 23 participants received teplizumab, with 20 receiving the full 14 day treatment course. Three participants discontinued teplizumab before day 14 due to prespecified protocol criteria (ESM Fig. 2). Two of the three received 1 day of teplizumab infusion and the third received 12 days of infusion. One additional participant received all 14 days of treatment but their second day of teplizumab infusion was delayed for 3 days due to the occurrence of a TEAE (haemoglobin decreased on day 2, considered possibly related to the drug). Seven of the 23 participants (30.4%) had at least one major deviation from the study protocol, although no deviation led to study withdrawal, as summarised in ESM results.

### Teplizumab safety profile

Over the course of the study, all 23 participants had one or more TEAE (Table 2). Most TEAEs were mild (grade 1) or moderate (grade 2), experienced by 95.7% and 73.9% of participants, respectively; 26.1% of participants had a grade 3 TEAE. No grade 4 or grade 5 TEAEs were reported. One participant experienced grade 1 CRS but this did not result in treatment discontinuation.

**Table 2:** Summary of TEAEs (enrolled population)

TEAEs	Teplizumab (*N*=23)
Any TEAE	23 (100)
TEAE during the dosing period and up to 28 days after last dose	23 (100)
Grade 1 TEAE	22 (95.7)
Grade 2 TEAE	17 (73.9)
Grade 3 TEAE	6 (26.1)
Grade 4 TEAE	0 (0)
Grade 5 TEAE	0 (0)
TEAE related to study drug	23 (100)
TEAE leading to study drug discontinuation	3 (13.0)
TEAE leading to study discontinuation	0 (0)
Serious TEAE	2 (8.7)
Serious TEAE related to study drug	2 (8.7)
Treatment-emergent AESI	4 (17.4)^a^
Treatment-emergent AESI related to study drug	3 (13.0)
Infusion-related TEAE	7 (30.4)
Injection site reaction TEAE	1 (4.3)
Death	0 (0)

Data are *n* (%)

A TEAE was defined as an adverse event that occurred after administration of the first dose of the study drug up to the end of the study. The severity of TEAEs was graded according to National Cancer Institute’s Common Terminology Criteria for Adverse Events (CTCAE) version 5.0 (https://dctd.cancer.gov/research/ctep-trials/for-sites/adverse-events). TEAEs related to the study drug were defined as TEAEs that were reported as possibly related, probably related or related to the study drug. Infusion-related and injection site reaction TEAEs were identified using a checkbox on the adverse event case report form

^a^One participant had lymphadenopathy that was associated by the investigator with a concomitant respiratory infection and not mononucleosis; consequently, the participant was not tested for mononucleosis and the event was not considered by the investigator to be an AESI

The most frequently reported TEAEs, both overall (occurring in ≥10% participants) and occurring during the dosing period and up to 28 days following the last dose, are provided in Table 3. With regard to overall TEAEs, most participants experienced gastrointestinal disorders (78.3%), infections (78.3%) and skin/subcutaneous tissue disorders (73.9%). The most frequently reported TEAEs occurring during the dosing period and up to 28 days after the last teplizumab dose were vomiting (52.2%), rash (43.5%), diarrhoea (30.4%), decreased lymphocyte count (30.4%), decreased white blood cell count (26.1%) and maculo-papular rash (26.1%). These TEAEs were consistent with the TEAEs deemed to be related to teplizumab (ESM Table 1). Episodes of vomiting were typically limited to one day but may have occurred on multiple days during and around the time of infusion. Ondansetron was used prophylactically or for treatment of vomiting in some cases.

**Table 3:** Incidence of TEAEs during dosing and up to 28 days after the last dose and incidence of overall TEAEs (occurring in ≥10% of participants) by system organ class and preferred term (enrolled population)

System organ class and preferred term	Incidence	
	TEAEs during dosing and up to day 28 after last dose^a^	All TEAEs^b^
Any TEAE	23 (100)	23 (100)
Gastrointestinal disorders	18 (78.3)	18 (78.3)
Vomiting	12 (52.2)	12 (52.2)
Diarrhoea	7 (30.4)	7 (30.4)
Nausea	5 (21.7)	6 (26.1)
Abdominal pain, upper	4 (17.4)	4 (17.4)
Constipation	1 (4.3)	4 (17.4)
Infections and infestations	6 (26.1)	18 (78.3)
Upper respiratory tract infection	2 (8.7)	12 (52.2)
Otitis media	1 (4.3)	4 (17.4)
Pharyngitis, streptococcal	1 (4.3)	3 (13.0)
Skin and subcutaneous tissue disorders	17 (73.9)	17 (73.9)
Rash	10 (43.5)	10 (43.5)
Rash, maculo-papular	6 (26.1)	6 (26.1)
Pruritus	4 (17.4)	5 (21.7)
Investigations	15 (65.2)	15 (65.2)
Lymphocyte count decreased	7 (30.4)	7 (30.4)
White blood cell count decreased	6 (26.1)	6 (26.1)
Haematocrit decreased	5 (21.7)	5 (21.7)
Blood bicarbonate decreased	2 (8.7)	4 (17.4)
Eosinophil count decreased	4 (17.4)	4 (17.4)
Alanine aminotransferase increased	3 (13.0)	3 (13.0)
Aspartate aminotransferase increased	3 (13.0)	3 (13.0)
Blood lactate dehydrogenase increased	3 (13.0)	3 (13.0)
Haemoglobin decreased	2 (8.7)	3 (13.0)
Mean cell haemoglobin decreased	2 (8.7)	3 (13.0)
General disorders and administration site conditions	8 (34.8)	12 (52.2)
Pyrexia	3 (13.0)	6 (26.1)
Fatigue	4 (17.4)	4 (17.4)
Respiratory, thoracic and mediastinal disorders	7 (30.4)	11 (47.8)
Cough	2 (8.7)	5 (21.7)
Nasal congestion	1 (4.3)	3 (13.0)
Blood and lymphatic system disorders	7 (30.4)	8 (34.8)
Anaemia	4 (17.4)	4 (17.4)
Lymphopenia	3 (13.0)	3 (13.0)
Injury, poisoning and procedural complications	6 (26.1)	8 (34.8)
Vascular access site pain	3 (13.0)	3 (13.0)
Metabolism and nutritional disorders	5 (21.7)	8 (34.8)
Decreased appetite	3 (13.0)	3 (13.0)
Nervous system disorders	3 (13.0)	6 (26.1)
Headache	3 (13.0)	5 (21.7)
Vascular disorders	4 (17.4)	5 (21.7)
Immune system disorders	1 (4.3)	3 (13.0)
Psychiatric disorders	2 (8.7)	3 (13.0)

Data are *n* (%)

With regard to incidence, participants with multiple events were counted only once for each preferred term and system organ class category. A TEAE was defined as an adverse event that occurred after administration of the first dose of the study drug up to the end of the study

^a^TEAEs occurring during the teplizumab dosing period and up to 28 days after the final dose were investigated to assess events occurring contemporaneously or shortly after treatment

^b^All TEAEs include TEAEs that occurred during dosing and up to day 28 after the last dose as well as those occurring after day 28

System organ classes and preferred terms were based on Medical Dictionary for Regulatory Activities (MedDRA) version 26.0 (https://www.meddra.org/)

Three participants (13.0%) had one or more TEAE leading to study drug discontinuation and these were considered to be AESIs. One participant discontinued teplizumab treatment due to anaemia on day 2 (considered to be related to the drug); this participant recovered spontaneously a few days later. Another participant discontinued treatment due to elevated alanine aminotransferase and aspartate aminotransferase levels on day 2 (considered to be probably related to the drug); values returned to the normal range within a few days. A third participant with a medical history of eczema and asthma discontinued treatment due to hospitalisation for a maculo-papular rash on day 11 (grade 3, considered to be related to the drug) and deep vein thrombosis at the site of the PICC line on day 13 (grade 2, non-occlusive; considered to be related to the PICC line placement and not to the drug); the participant recovered from both events with supportive treatment with enoxaparin and prophylactic antibiotics initially.

In total, two participants (8.7%) experienced serious TEAEs (two serious TEAEs each). As described above, one participant (aged 5.9 years) experienced a maculo-papular rash and a deep vein thrombosis at the site of the PICC line insertion. These two serious TEAEs were the only serious TEAEs reported during the dosing period and up to 28 days after the last dose. Another participant (aged 6.2 years) with a medical history of asthma and recurrent hospitalisations for asthma exacerbations was diagnosed with a glioneuronal tumour following symptoms of upper back/posterior neck pain approximately 12 months after the first teplizumab dose (grade 3, deemed possibly related to teplizumab by the investigator [no prior imaging of the spinal cord was available] but deemed not related to teplizumab by an independent external paediatric neuro-oncologist). The histopathology report determined that the tumour was low grade. This same participant experienced an asthmatic crisis (grade 3, deemed not related to teplizumab) at approximately 16 months.

Haematology data at week 52 were available for 13 of the 15 participants who reached the cut-off date for the interim analysis. At week 52, the mean change from baseline in lymphocyte count was −0.588 × 10^9^ cells/l and the mean change in lymphocyte percentage of total white blood cells was −4.19%. These decreases were not considered clinically relevant and were consistent with the known safety profile of teplizumab.

No participants experienced vital sign abnormalities that were considered serious TEAEs or that led to teplizumab discontinuation.

### Pharmacokinetics and pharmacodynamics

Mean serum teplizumab concentrations increased over time in the pharmacokinetics population, peaking at day 14 following infusion (Fig. 1). By day 28, the mean serum teplizumab concentration decreased to values close to the lower limit of quantification of 2.5 ng/ml.

In the teplizumab pharmacodynamics population, two participants were excluded because dosing was discontinued prior to day 9 and no post-baseline samples were available. The mean percentage of CD3 occupancy by teplizumab was 78.4% (SD 9.8) at day 9 post infusion. The mean percentage of circulating CD3-expressing T cells among total T cells decreased from 92.58% (SD 4.93) at baseline to 85.50% (SD 9.91) at day 9 post infusion. These results are consistent with the mechanism of action of teplizumab. Consistent with the known effect of teplizumab, the mean lymphocyte count transiently decreased during the treatment course, with the nadir observed around day 5, and returned to baseline values by day 28.

### Immunogenicity

**Fig. 2:**
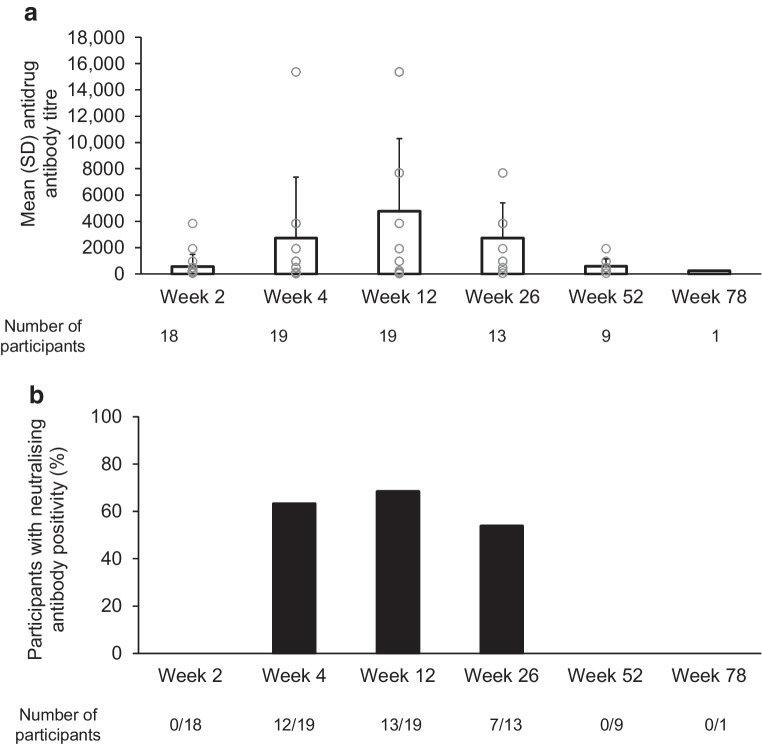
Antidrug antibody titres (**a**) and percentage of participants with NAbs (**b**) (immunogenicity population) post baseline. The immunogenicity population was defined as all enrolled participants who provided one or more post-baseline antidrug antibody sample. Antidrug antibody titres and NAb status were summarised for participants with a positive antidrug antibody status at each visit. Unfilled circles (**a**) show individual participant data

At baseline, antidrug antibody data were available for 21 participants, all of whom were antibody negative. Post-baseline antidrug antibody data were available for 20 participants, as one participant received only one dose of teplizumab before permanently discontinuing treatment. At week 2 (day 14), 18 participants (90% of those with antidrug antibody sample data) were antidrug antibody positive. Antidrug antibody titres increased from week 2 up to week 12 and then decreased (Fig. 2a). Antidrug antibody-positive blood samples were further tested for NAbs targeting teplizumab. At week 2 (day 14), none of the 18 participants with antidrug antibody positivity had detectable NAbs. NAb positivity was detected in over half of participants with antidrug antibody positivity at weeks 4, 12 and 26 (Fig. 2b). At week 52, no participants (0/9) had NAb positivity.

Participants were stratified into visit-specific quartiles based on antidrug antibody titre to determine if the presence of antidrug antibodies influenced teplizumab serum concentrations. Based on this antidrug antibody titre quartile evaluation, antidrug antibodies did not have an observable effect on teplizumab pharmacokinetics (data not shown). However, NAb-positive participants had a lower mean teplizumab serum concentration (9.76 [SD 11.72]) than NAb-negative participants (35.42 [SD 16.16]) at day 28.

### Time to development of stage 3 type 1 diabetes and HbA_1c_

**Fig. 3:**
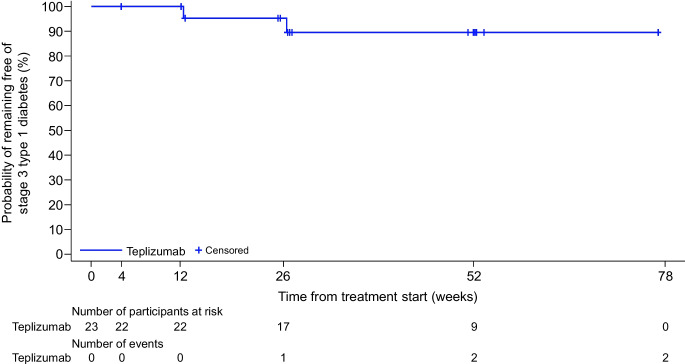
Elapsed time from study treatment initiation to the development of stage 3 type 1 diabetes in the enrolled population, defined as all participants who received one or more doses of teplizumab

Two participants met criteria for progression to stage 3 type 1 diabetes by the time of the interim analysis (median follow-up duration for the enrolled population was 51.9 weeks) (Fig. 3). The first participant (aged 1.7 years; GADA, IAA, IA-2A and ZnT8A positivity; HbA_1c_ 43 mmol/mol [6.1%] at baseline; one sibling with type 1 diabetes) was diagnosed with stage 3 type 1 diabetes at 12.4 weeks after the treatment start date, due to an HbA_1c_ level of 49 mmol/mol (6.6%). The second participant (aged 6.2 years; GADA, IAA and ZnT8A positivity; HbA_1c_ 43 mmol/mol [6.1%] at baseline; no relatives with type 1 diabetes) was diagnosed with stage 3 type 1 diabetes at 26.4 weeks after the treatment start date, due to a fasting plasma glucose level of 7.9 mmol/l (142 mg/dl) and an HbA_1c_ level of 55 mmol/mol (7.2%). Based on additional search criteria for the diagnosis of stage 3 type 1 diabetes, one participant experienced a TEAE of hyperglycaemia; the investigator evaluated the TEAE with additional testing and confirmed that the participant had not progressed to stage 3 type 1 diabetes. The estimated probability that a participant remained free of progression to stage 3 type 1 diabetes was 89.6% (95% CI 64.3%, 97.3%) at the time of the interim analysis.

HbA_1c_ values at baseline were available for all 23 treated participants; at week 52, HbA_1c_ data were available for all 15 participants with ≥52 weeks of follow-up at the time of the interim analysis. No clinically meaningful change from baseline was observed in HbA_1c_ levels throughout the course of the study up to week 52 (week 12: mean change +1.1 mmol/mol, SD 3.2 [+0.10%, SD 0.29]; week 26: mean change +0.8 mmol/mol, SD 3.9 [+0.07%, SD 0.36]; week 52: mean change +2.0 mmol/mol, SD 2.7 [+0.18%, SD 0.25]). HbA_1c_ levels did not differ across antidrug antibody titre quartiles, nor between NAb-positive and NAb-negative participants (data not shown).

## Discussion

This interim analysis of the PETITE-T1D study provides the first assessment of the safety and tolerability of teplizumab in children aged <8 years with stage 2 type 1 diabetes, with secondary measures including the immunogenicity, pharmacokinetics and pharmacodynamics of teplizumab and participant progression to stage 3 type 1 diabetes. The most frequent TEAEs considered to be related to teplizumab were vomiting, rash, decreased lymphocyte count, decreased white blood cell count and diarrhoea. One participant had a serious TEAE (maculo-papular rash) considered to be related to teplizumab, although this participant had a medical history of eczema and asthma, and also a serious TEAE related to the PICC line placement (deep vein thrombosis) that was deemed not to be related to teplizumab. Three participants had TEAEs that led to discontinuation of teplizumab (maculo-papular rash in one participant, anaemia in another participant and increased liver enzymes in another participant), although no other haematology, clinical chemistry or vital sign abnormalities were considered serious or led to teplizumab discontinuation. CRS occurred in one participant (4.3%) and was mild, with the rate comparable to the rates of CRS detected in the Phase 3 PROTECT trial including children and adolescents aged ≥8 years of age and in an integrated analysis of five clinical trials (8.8% and 5.8%, respectively) [24, 25], suggesting that CRS is an uncommon TEAE following teplizumab treatment. Overall, the safety findings are consistent with the known safety profile of teplizumab and no new safety risks were identified [24, 25]. Of note, the reported rate of vomiting in the PETITE-T1D trial (52.2%) was higher than that reported in the PROTECT trial (31.8%) [25] and in the integrated analysis of five clinical trials (13.9%) (ESM Table 2) [24]. The younger children in this study may have been less likely to communicate symptoms of nausea to investigators and therefore may have been less likely to receive ondansetron pre-emptively to avoid vomiting. Further investigation would be needed to determine if higher levels of cytokines were released in the younger population in the current study. In this study, vomiting was not associated with other symptoms of CRS or with a viral syndrome.

Regarding the participant with a glioneuronal tumour diagnosis approximately 12 months after teplizumab treatment, it was not possible to establish whether the glioneuronal tumour was present before starting treatment because no imaging of the spinal cord had been performed before diagnosis. Low-grade glioneuronal tumour is an extremely rare type of paediatric central nervous system tumour that occurs sporadically and has not been associated with T cell therapies or immune-associated diseases. An external independent expert in paediatric neuro-oncology assessed the event as not related to teplizumab; this expert considered that the tumour was likely to have been present at the time of the participant’s enrolment in the trial, considering its large size at diagnosis and the extremely slow growth rate of such tumours. However, an effect of teplizumab on the growth rate of the tumour cannot be excluded.

As seen in previous studies in participants aged ≥8 years, immunogenicity of teplizumab was observed, with mean antidrug antibody titres peaking at week 12 and subsequently declining up to week 52. In the pivotal TN-10 trial [31], antidrug antibodies were identified in approximately 57% of participants treated with teplizumab [21]. Overall, 90% of participants in the present study showed antidrug antibody positivity at day 14. The percentage of NAb-positive participants peaked at week 12, but no participants were NAb positive at week 52.

The teplizumab mean serum concentration peaked at day 14, consistent with a previous report of teplizumab administered over a 14 day course [34]. Although antidrug antibody titres were not associated with teplizumab concentrations, participants with NAb positivity showed lower concentrations of teplizumab than those without NAb positivity. These findings are similar to those of the Phase 3 Protégé trial, which reported that teplizumab pharmacokinetics were influenced by the development of antidrug antibodies [35]. Notably, in the Protégé trial, no relationship between antidrug antibody levels and change in C-peptide AUC was apparent, despite the influence of antidrug antibodies on pharmacokinetics [35].

In the present study, the majority of CD3 complexes (mean 78.4%) were occupied by teplizumab on day 9 post infusion, providing evidence that teplizumab bound to its target [21, 35]. Furthermore, the percentage of circulating CD3^+^ T cells among total T cells decreased from baseline to day 9, consistent with the mechanism of action of teplizumab, which is proposed to involve internalisation of CD3/T cell receptors from the cell surface of activated T cells [21, 35, 36]. Other effects of teplizumab include a transient decline in circulating lymphocytes due to margination and not depletion [24, 31, 37, 38]. Consistent with this, there was a transient decrease in lymphocyte count that resolved by day 28, consistent with data from the PROTECT trial [25]. In the present study, the incidences of decreased lymphocyte count and lymphopenia were 30.4% and 13.0%, respectively, similar to those reported in the PROTECT trial (33.6% and 23.0%, respectively) [25] but lower than what was reported in the integrated analysis of five trials (79.9% of those treated with teplizumab had lymphopenia) (ESM Table 2) [24]. The percentage of participants with a decreased white blood cell count was 26.1%, which was similar to that in the PROTECT trial (24.4%) [25].

By the cut-off date for the interim analysis, two participants developed stage 3 type 1 diabetes. In the TN-10 trial [31], the estimated probability of remaining free of progression to stage 3 type 1 diabetes at 1 year among participants aged ≥8 years was 0.93 (95% CI 0.80, 0.98) for those receiving teplizumab and 0.65 (95% CI 0.46, 0.79) for those receiving placebo. Results from the interim analysis of PETITE-T1D were similar, with an estimated probability of remaining free of progression to stage 3 type 1 diabetes of 0.90 (95% CI 0.64, 0.97). Additionally, an observational study of untreated children screened for early-stage type 1 diabetes at <11 years of age suggested that the probability of remaining free of progression to stage 3 for those with stage 2 type 1 diabetes (*n*=87) was 0.76 at 1 year [39] (based on the reported 1 year risk of progression to stage 3 of 28% [39]; under an exponential assumption, the corresponding probability of remaining progression free at 1 year is approximately 0.76). This observational study described the progression of disease in children who did not receive teplizumab, helping to contextualise the findings from participants exposed to the treatment. However, direct comparisons cannot be made across studies given differences in baseline characteristics. Rate of progression to stage 3 type 1 diabetes is associated with multiple factors including age at seroconversion, with younger ages at seroconversion associated with faster progression [7, 26, 39, 40]. As an example, an analysis of TrialNet Pathway to Prevention data found that the 5 year rates of progression from multiple autoantibody positivity to stage 3 type 1 diabetes were 35%, 22% and 15% in individuals who seroconverted at <12 years of age, ≥12 years and >18 years, respectively [40]. Surveillance of progression to stage 3 type 1 diabetes is ongoing in the children enrolled in the PETITE-T1D study.

We acknowledge the following study limitations. First, PETITE-T1D was a small, single-arm study that aimed to investigate the safety profile of teplizumab and therefore efficacy analyses were not a study objective and are not possible. We also note that the study cohort was confined almost exclusively to a White population, despite our best efforts to recruit a diverse study population. Although the sample size was small (23 participants, with 20 completing treatment), it was determined that 15 participants completing treatment would provide an 80% probability of observing at least one adverse event if the true population rate was 8‒10%. An additional limitation of the study is that 87% of participants came from families with an index case of type 1 diabetes, whereas approximately 90% of those with new-onset type 1 diabetes in the general population have no first-degree relative with the disease [41, 42]. Lastly, the determination of progression to stage 3 type 1 diabetes was based on ADA criteria, which include glycaemic levels, OGTTs and HbA_1c_, and did not include protocol-defined scheduled OGTTs, which are highly sensitive tests for the early detection of stage 3 type 1 diabetes [33, 43]. Continuous glucose monitoring metric analyses will be conducted at the end of the study. Serological outcomes related to childhood vaccination, including response to diphtheria, tetanus and acellular pertussis (DTaP) vaccination, will be analysed at the end of the study.

In conclusion, teplizumab was well tolerated and demonstrated a safety and tolerability profile consistent with that observed in participants aged ≥8 years in clinical trials and post-marketing experience to date. The results presented are those of a planned interim analysis. Comprehensive study data will be analysed on completion of the 104 week study.

## Supplementary Information

Below is the link to the electronic supplementary material.

ESM (PDF 439 KB)

## Data Availability

Qualified researchers may request access to data. Further details on Sanofi’s data sharing criteria, eligible studies and process for requesting access can be found at: https://www.vivli.org.

## Abbreviations

AESI: Adverse event of special interest
CRS: Cytokine release syndrome
FDA: Food and Drug Administration
NAb: Neutralising antibody
PICC: Peripherally inserted central catheter
TEAE: Treatment-emergent adverse event

## Authors’ relationships and activities

SEG has served on advisory boards for Abata Therapeutics, Genentech, GentiBio, Eli Lilly, Provention Bio, SAB Biotherapeutics, Sana Biotechnology, Sanofi and Shoreline Biosciences; has received support from the National Institutes for Health (NIH), Provention Bio and Sanofi for roles as an investigator in clinical trials; and serves on data and safety monitoring boards for Diamyd Medical, Breakthrough T1D (formerly known as JDRF) and INNODIA. KS has served on advisory boards for Provention Bio and Sanofi and has received support from the NIH, Breakthrough T1D, Sanofi and Provention Bio for roles as an investigator in clinical trials. JLS consults for Abbott Diabetes, Insulet, Medscape, Medtronic Diabetes, Vertex and Ypsomed; has served on advisory boards for Cecelia Health, Insulet, MannKind, Medtronic Diabetes, StartUp Health T1D Moonshot and Vertex; and has received research support from Abbott Diabetes, Dexcom, Breakthrough T1D, Insulet, Medtronic, NIH and Provention Bio. SBL has consulted for Sanofi. TQ is a consultant for BioMarin, Janssen Research & Development, Merck, Pfizer, Provention Bio and Sanofi and is a Clinical Trial Principal Investigator at the Buffalo site for Ascendis, BioMarin, Janssen, OPKO Biologics, Pfizer, Provention Bio and Sanofi. WER has received grants or contracts from NIH and Provention Bio. BS has consulted for Provention Bio. SMW has received support from NIH, Provention Bio and Sanofi for roles as an investigator in clinical trials. LK, EN, WS and DM are employees of Sanofi and may hold stock in the company. IL is an employee of Cytel and was serving as a vendor to Sanofi at the time of the study. LAD has served on advisory boards for Abata Therapeutics, Tandem and Biomea Fusion; has received research support from NIH, Lilly, MannKind, Provention Bio, Sanofi and Zealand for roles as an investigator in clinical trials; has received study supplies from Dexcom; and serves on data safety monitoring boards for Lilly and Merck for non-diabetes indications.

## References

[1] Menke A, Orchard TJ, Imperatore G, Bullard KM, Mayer-Davis E, Cowie CC (2013) The prevalence of type 1 diabetes in the United States. Epidemiology 24(5):773–774. 10.1097/EDE.0b013e31829ef01a 23903880 10.1097/EDE.0b013e31829ef01a PMC4562437

[2] Tonnies T, Brinks R, Isom S et al (2023) Projections of type 1 and type 2 diabetes burden in the U.S. population aged <20 years through 2060: the SEARCH for diabetes in youth study. Diabetes Care 46(2):313–320. 10.2337/dc22-0945 36580405 10.2337/dc22-0945 PMC9887625

[3] Divers J, Mayer-Davis EJ, Lawrence JM et al (2020) Trends in incidence of type 1 and type 2 diabetes among youths - selected counties and Indian reservations, United States, 2002–2015. MMWR Morb Mortal Wkly Rep 69(6):161–165. 10.15585/mmwr.mm6906a3 32053581 10.15585/mmwr.mm6906a3 PMC7017961

[4] Phillip M, Achenbach P, Addala A et al (2024) Consensus guidance for monitoring individuals with islet autoantibody-positive pre-stage 3 type 1 diabetes. Diabetes Care 47(8):1276–1298. 10.2337/dci24-0042 38912694 10.2337/dci24-0042 PMC11381572

[5] Riley WJ, Maclaren NK, Krischer J et al (1990) A prospective study of the development of diabetes in relatives of patients with insulin-dependent diabetes. N Engl J Med 323(17):1167–1172. 10.1056/NEJM199010253231704 2215594 10.1056/NEJM199010253231704

[6] Wenzlau JM, Juhl K, Yu L et al (2007) The cation efflux transporter ZnT8 (Slc30A8) is a major autoantigen in human type 1 diabetes. Proc Natl Acad Sci U S A 104(43):17040–17045. 10.1073/pnas.0705894104 17942684 10.1073/pnas.0705894104 PMC2040407

[7] Ziegler AG, Rewers M, Simell O et al (2013) Seroconversion to multiple islet autoantibodies and risk of progression to diabetes in children. JAMA 309(23):2473–2479. 10.1001/jama.2013.6285 23780460 10.1001/jama.2013.6285 PMC4878912

[8] Monaghan M, Bryant BL, Inverso H, Moore HR, Streisand R (2022) Young children with type 1 diabetes: recent advances in behavioral research. Curr Diab Rep 22(6):247–256. 10.1007/s11892-022-01465-0 35435615 10.1007/s11892-022-01465-0 PMC9013975

[9] Azimi T, Johnson J, Campbell SM, Montesanti S (2024) Caregiver burden among parents of children with type 1 diabetes: a qualitative scoping review. Heliyon 10(6):e27539. 10.1016/j.heliyon.2024.e27539 38524615 10.1016/j.heliyon.2024.e27539 PMC10958210

[10] Demeterco-Berggren C, Ebekozien O, Noor N et al (2022) Factors associated with achieving target A1C in children and adolescents with type 1 diabetes: findings from the T1D exchange quality improvement collaborative. Clin Diabetes 41(1):68–75. 10.2337/cd22-0073 36714245 10.2337/cd22-0073 PMC9845079

[11] Foster NC, Beck RW, Miller KM et al (2019) State of type 1 diabetes management and outcomes from the T1D exchange in 2016–2018. Diabetes Technol Ther 21(2):66–72. 10.1089/dia.2018.0384 30657336 10.1089/dia.2018.0384 PMC7061293

[12] Cardona-Hernandez R, Schwandt A, Alkandari H et al (2021) Glycemic outcome associated with insulin pump and glucose sensor use in children and adolescents with type 1 diabetes. Data from the international pediatric registry SWEET. Diabetes Care 44(5):1176–1184. 10.2337/dc20-1674 33653821 10.2337/dc20-1674

[13] Sundberg F, deBeaufort C, Krogvold L et al (2022) ISPAD clinical practice consensus guidelines 2022: managing diabetes in preschoolers. Pediatr Diabetes 23(8):1496–1511. 10.1111/pedi.13427 36537520 10.1111/pedi.13427 PMC10108244

[14] Foland-Ross LC, Tong G, Mauras N et al (2020) Brain function differences in children with type 1 diabetes: a functional MRI study of working memory. Diabetes 69(8):1770–1778. 10.2337/db20-0123 32471809 10.2337/db20-0123 PMC7372069

[15] Mauras N, Buckingham B, White NH et al (2021) Impact of type 1 diabetes in the developing brain in children: a longitudinal study. Diabetes Care 44(4):983–992. 10.2337/dc20-2125 33568403 10.2337/dc20-2125 PMC7985430

[16] Suput Omladic J, Slana Ozimic A, Vovk A et al (2020) Acute hyperglycemia and spatial working memory in adolescents with type 1 diabetes. Diabetes Care 43(8):1941–1944. 10.2337/dc20-0171 32471909 10.2337/dc20-0171 PMC7372055

[17] Schwartz DD, Wasserman R, Powell PW, Axelrad ME (2014) Neurocognitive outcomes in pediatric diabetes: a developmental perspective. Curr Diab Rep 14(10):533. 10.1007/s11892-014-0533-x 25142718 10.1007/s11892-014-0533-x PMC4864497

[18] Desrocher M, Rovet J (2004) Neurocognitive correlates of type 1 diabetes mellitus in childhood. Child Neuropsychol 10(1):36–52. 10.1076/chin.10.1.36.26241 14977514 10.1076/chin.10.1.36.26241

[19] Languren G, Montiel T, Julio-Amilpas A, Massieu L (2013) Neuronal damage and cognitive impairment associated with hypoglycemia: an integrated view. Neurochem Int 63(4):331–343. 10.1016/j.neuint.2013.06.018 23876631 10.1016/j.neuint.2013.06.018

[20] Hershey T, Perantie DC, Wu J, Weaver PM, Black KJ, White NH (2010) Hippocampal volumes in youth with type 1 diabetes. Diabetes 59(1):236–241. 10.2337/db09-1117 19833895 10.2337/db09-1117 PMC2797927

[21] Provention Bio Inc. (2025) Tzield. Highlights of prescribing information. Available from: https://products.sanofi.us/tzield/tzield.pdf. Accessed: 2 Oct 2025

[22] Lledo-Delgado A, Preston-Hurlburt P, Currie S et al (2024) Teplizumab induces persistent changes in the antigen-specific repertoire in individuals at risk for type 1 diabetes. J Clin Invest 134(18):e177492. 10.1172/JCI177492 39137044 10.1172/JCI177492 PMC11405034

[23] Sims EK, Bundy BN, Stier K et al (2021) Teplizumab improves and stabilizes beta cell function in antibody-positive high-risk individuals. Sci Transl Med 13(583):eabc8980. 10.1126/scitranslmed.abc8980 33658358 10.1126/scitranslmed.abc8980 PMC8610022

[24] Herold KC, Gitelman SE, Gottlieb PA, Knecht LA, Raymond R, Ramos EL (2023) Teplizumab: a disease-modifying therapy for type 1 diabetes that preserves beta-cell function. Diabetes Care 46(10):1848–1856. 10.2337/dc23-0675 37607392 10.2337/dc23-0675 PMC10545553

[25] Ramos EL, Dayan CM, Chatenoud L et al (2023) Teplizumab and beta-cell function in newly diagnosed type 1 diabetes. N Engl J Med 389(23):2151–2161. 10.1056/NEJMoa2308743 37861217 10.1056/NEJMoa2308743

[26] Frohnert BI, Ghalwash M, Li Y et al (2023) Refining the definition of stage 1 type 1 diabetes: an ontology-driven analysis of the heterogeneity of multiple islet autoimmunity. Diabetes Care 46(10):1753–1761. 10.2337/dc22-1960 36862942 10.2337/dc22-1960 PMC10516254

[27] Herold KC, Hagopian W, Auger JA et al (2002) Anti-CD3 monoclonal antibody in new-onset type 1 diabetes mellitus. N Engl J Med 346(22):1692–1698. 10.1056/NEJMoa012864 12037148 10.1056/NEJMoa012864

[28] Herold KC, Gitelman SE, Ehlers MR et al (2013) Teplizumab (anti-CD3 mAb) treatment preserves C-peptide responses in patients with new-onset type 1 diabetes in a randomized controlled trial: metabolic and immunologic features at baseline identify a subgroup of responders. Diabetes 62(11):3766–3774. 10.2337/db13-0345 23835333 10.2337/db13-0345 PMC3806618

[29] Herold KC, Gitelman SE, Willi SM et al (2013) Teplizumab treatment may improve C-peptide responses in participants with type 1 diabetes after the new-onset period: a randomised controlled trial. Diabetologia 56(2):391–400. 10.1007/s00125-012-2753-4 23086558 10.1007/s00125-012-2753-4 PMC3537871

[30] Sherry N, Hagopian W, Ludvigsson J et al (2011) Teplizumab for treatment of type 1 diabetes (Protege study): 1-year results from a randomised, placebo-controlled trial. Lancet 378(9790):487–497. 10.1016/S0140-6736(11)60931-8 21719095 10.1016/S0140-6736(11)60931-8 PMC3191495

[31] Herold KC, Bundy BN, Long SA et al (2019) An Anti-CD3 antibody, teplizumab, in relatives at risk for type 1 diabetes. N Engl J Med 381(7):603–613. 10.1056/NEJMoa1902226 31180194 10.1056/NEJMoa1902226 PMC6776880

[32] American Diabetes Association Professional Practice Committee (2022) 2. Classification and diagnosis of diabetes: standards of medical care in diabetes-2022. Diabetes Care 45(Suppl 1):S17–S38. 10.2337/dc22-S002 34964875 10.2337/dc22-S002

[33] American Diabetes Association Professional Practice Committee (2025) 2. Diagnosis and classification of diabetes: standards of care in diabetes-2025. Diabetes Care 48(Supplement_1):S27–S49. 10.2337/dc25-S002 39651986 10.2337/dc25-S002 PMC11635041

[34] Daifotis AG, Koenig S, Chatenoud L, Herold KC (2013) Anti-CD3 clinical trials in type 1 diabetes mellitus. Clin Immunol 149(3):268–278. 10.1016/j.clim.2013.05.001 23726024 10.1016/j.clim.2013.05.001

[35] Hagopian W, Ferry RJ Jr, Sherry N et al (2013) Teplizumab preserves C-peptide in recent-onset type 1 diabetes: two-year results from the randomized, placebo-controlled Protege trial. Diabetes 62(11):3901–3908. 10.2337/db13-0236 23801579 10.2337/db13-0236 PMC3806608

[36] Kuhn C, Weiner HL (2016) Therapeutic anti-CD3 monoclonal antibodies: from bench to bedside. Immunotherapy 8(8):889–906. 10.2217/imt-2016-0049 27161438 10.2217/imt-2016-0049

[37] Herold KC, Gitelman S, Greenbaum C et al (2009) Treatment of patients with new onset Type 1 diabetes with a single course of anti-CD3 mAb Teplizumab preserves insulin production for up to 5 years. Clin Immunol 132(2):166–173. 10.1016/j.clim.2009.04.007 19443276 10.1016/j.clim.2009.04.007 PMC2735402

[38] Waldron-Lynch F, Henegariu O, Deng S et al (2012) Teplizumab induces human gut-tropic regulatory cells in humanized mice and patients. Sci Transl Med 4(118):118ra112. 10.1126/scitranslmed.3003401 10.1126/scitranslmed.3003401 PMC4131554 22277969

[39] Weiss A, Zapardiel-Gonzalo J, Voss F et al (2022) Progression likelihood score identifies substages of presymptomatic type 1 diabetes in childhood public health screening. Diabetologia 65(12):2121–2131. 10.1007/s00125-022-05780-9 36028774 10.1007/s00125-022-05780-9 PMC9630406

[40] Jacobsen LM, Bocchino L, Evans-Molina C et al (2020) The risk of progression to type 1 diabetes is highly variable in individuals with multiple autoantibodies following screening. Diabetologia 63(3):588–596. 10.1007/s00125-019-05047-w 31768570 10.1007/s00125-019-05047-w PMC7229995

[41] Turtinen M, Harkonen T, Parkkola A, Ilonen J, Knip M, Finnish Pediatric Diabetes Register (2019) Characteristics of familial type 1 diabetes: effects of the relationship to the affected family member on phenotype and genotype at diagnosis. Diabetologia 62(11):2025–2039. 10.1007/s00125-019-4952-8 31346657 10.1007/s00125-019-4952-8 PMC6805821

[42] Karges B, Prinz N, Placzek K et al (2021) A comparison of familial and sporadic type 1 diabetes among young patients. Diabetes Care 44(5):1116–1124. 10.2337/dc20-1829 33824143 10.2337/dc20-1829

[43] Sakaguchi K, Takeda K, Maeda M et al (2016) Glucose area under the curve during oral glucose tolerance test as an index of glucose intolerance. Diabetol Int 7(1):53–58. 10.1007/s13340-015-0212-4 30603243 10.1007/s13340-015-0212-4 PMC6214468

