# Supplementary material for: Safety and pharmacokinetics of teplizumab in children less than 8 years of age with stage 2 type 1 diabetes
Source: Diabetologia. 2025 Nov 6;69(2):330–42. doi: 10.1007/s00125-025-06586-1 (PMC12779737; doi:10.1007/s00125-025-06586-1)
Supplement: Supplementary file 1 — ESM (PDF 439 KB) [file 125_2025_6586_MOESM1_ESM.pdf]

Safety and pharmacokinetics of teplizumab in children less than 8 years of age with stage 2 type 1 diabetes

Stephen E. Gitelman; Kimber Simmons; Jennifer L. Sherr; Steven B. Leichter; Teresa Quattrin; William E. Russell; Bhuvana Sunil; Steven M. Willi; Laura A. Knecht; Elisabeth Niemoeller; Idlir Licaj; Wolfgang Schmider; Diana Miller; Linda A. DiMeglio

ELECTRONIC SUPPLEMENTARY MATERIAL (ESM)

ESM Methods

Adverse events of special interest

Prespecified adverse events of special interest included: all ≥ grade 3 infections, acute mononucleosis-like illness (including clinical Epstein–Barr virus and cytomegalovirus infections and reactivations), malignancies including lymphomas, severe hypoglycaemic episodes, ≥ grade 3 liver function abnormalities, ≥ grade 3 thrombocytopenia, ≥ grade 4 allergic/hypersensitivity reaction, ≥ grade 3 rash, ≥ grade 4 cytokine release syndrome, ≥ grade 3 neutropenia (on two consecutive evaluations performed on different days), lymphocyte count <500/mm^3 for 7 days or longer, pregnancy of a female participant enrolled in the study, and symptomatic overdose (serious or not). Additionally, there were no adverse events of special interest of ≥ grade 3 allergic/hypersensitivity reaction or ≥ grade 3 cytokine release syndrome.

Timing of secondary endpoint assessments

The secondary endpoints and time points included for the interim analysis were serum concentrations of teplizumab pre- and post-infusion (post-infusion sample collections occurred approximately 1 hour after the end of the infusions) at Days 1, 4, 6, 9, 14; serum concentrations of teplizumab pre-infusion on Day 12; serum concentrations of teplizumab on Day 28 (Week 4); antidrug antibody titres and presence of neutralising antibodies at Day 1 and Weeks 2, 4, 12, 26, and 52; and CD3 receptor occupancy on Day 1 (pre-dose) and Day 9 (post-dose).

ESM Results

Protocol deviations

Major protocol deviations were deviations that may have impacted completeness, accuracy, and/or reliability of the study data or that may have significantly affected a participant’s rights, safety, or well-being. The following major protocol deviations occurred in a total of seven participants.

One participant was administered teplizumab despite haemoglobin values ≤10 g/dl (a criterion for treatment discontinuation) on Days 5, 9, 12, and 14 per the investigator’s assessment that the value was not a significant deviation from their baseline of 10.2 g/dl. For one participant, body surface area was not rounded to the hundredth place for dose calculation, resulting in an approximately 3% higher teplizumab dose than intended. One participant received both MMR and varicella vaccines roughly 5 months before the allowed date per the study protocol (12 months post-teplizumab infusion), despite appropriate education. For six

participants, deviations were due to the following: serum teplizumab concentrations not obtained, vital signs close to the infusion (pre- and/or post-infusion) not recorded, missing chemistry values, and hypotension during infusion not monitored.

ESM Table 1. Summary of TEAEs assessed as related to study treatment by preferred terms in ≥10% of participants (enrolled population)

**Preferred term**	**Incidence, n (%)**
Vomiting	11 (47.8)
Rash	10 (43.5)
Lymphocyte count decreased	7 (30.4)
White blood cell count decreased	6 (26.1)
Diarrhoea	6 (26.1)
Rash maculo-papular	5 (21.7)
Nausea	4 (17.4)
Pruritus	4 (17.4)
Haematocrit decreased	4 (17.4)
Eosinophil count increased	4 (17.4)
Anaemia	4 (17.4)
Abdominal pain upper	3 (13.0)
Upper respiratory tract infection	3 (13.0)
Alanine aminotransferase increased	3 (13.0)
Aspartate aminotransferase increased	3 (13.0)
Haemoglobin decreased	3 (13.0)
Pyrexia	3 (13.0)
Fatigue	3 (13.0)
Lymphopenia	3 (13.0)
Decreased appetite	3 (13.0)
Headache	3 (13.0)

Preferred terms were based on Medical Dictionary for Regulatory Activities (MedDRA) version 26.0 (https://www.meddra.org/). A TEAE is defined as an AE that occurs after the first dose of study drug through the end of the study. TEAEs that were related to the study drug were defined as TEAEs that were reported as possibly related, probably related, or related to study drug. If the assessment of the relationship to study drug was missing for an AE, this AE was assumed to be related to study drug. Participants with multiple events are counted only once for each preferred term. Percentages are based on the number of participants in the enrolled population. AE, adverse event; TEAE, treatment-emergent adverse event.

ESM Table 2. Incidence of TEAEs in PETITE-T1D, an integrated analysis of five trials [24], and the PROTECT trial [25] occurring in ≥10% of participants treated with teplizumab in any of the three studies. Incidence is only reported if ≥10%

**Preferred term**	PETITE-T1D (N=23) n (%)	Integrated analysis, teplizumab (N=791) n (%)	PROTECT, teplizumab (N=217) n (%)
Abdominal pain	-	-	37 (17.1)
Abdominal pain, upper	4 (17.4)	-	31 (14.3)
Alanine aminotransferase increased	3 (13.0)	210 (26.5)	28 (12.9)
Anaemia	4 (17.4)	-	-
Aspartate aminotransferase increased	3 (13.0)	222 (28.1)	-
Blood alkaline phosphatase decreased	-	107 (13.5)	-
Blood bicarbonate decreased	4 (17.4)	303 (38.3)	
Blood calcium decreased	-	100 (12.6)	
Blood lactate dehydrogenase increased	3 (13.0)	-	-
Blood sodium decreased	-	129 (16.3)	-
Constipation	4 (17.4)	-	-
Cough	5 (21.7)	-	-
COVID-19	-	-	49 (22.6)
Decreased appetite	3 (13.0)	-	-
Diarrhoea	7 (30.4)	-	31 (14.3)
Eosinophil count decreased	4 (17.4)	-	-
Fatigue	4 (17.4)	80 (10.1)	22 (10.1)
Haematocrit decreased	5 (21.7)	-	-
Haemoglobin decreased	3 (13.0)	228 (28.8)	-
Headache	5 (21.7)	215 (27.2)	94 (43.3)
Hypocalcaemia	-	137 (17.3)	-
Hypoglycaemia	-	-	151 (69.9)
Hyponatremia	-	170 (21.5)	-
Leukopenia	-	501 (63.3)	26 (12.0)
Lymphocyte count decreased	7 (30.4)	-	73 (33.6)
Lymphopenia	3 (13.0)	632 (79.9)	50 (23.0)
Mean cell haemoglobin decreased	3 (13.0)	-	-

Nasal congestion	3 (13.0)	-	-
Nasopharyngitis	-	88 (11.1)	-
Nausea	6 (26.1)	155 (19.6)	92 (42.4)
Neutropenia	-	313 (39.6)	28 (12.9)
Neutrophil count decreased	-	-	33 (15.2)
Otitis media	4 (17.4)	-	-
Pharyngitis, streptococcal	3 (13.0)	-	-
Pruritus	5 (21.7)	118 (14.9)	-
Pyrexia	6 (26.1)	188 (23.8)	53 (24.4)
Rash	10 (43.5)	273 (34.5)	86 (39.6)
Rash, maculo-papular	6 (26.1)	-	29 (13.4)
Thrombocytopenia	-	172 (21.7)	-
Upper respiratory tract infection	12 (52.2)	150 (19.0)	44 (20.3)
Vascular access site pain	3 (13.0)	-	-
Vomiting	12 (52.2)	110 (13.9)	69 (31.8)
White blood cell count decreased	6 (26.1)	-	53 (24.4)

Dashes indicate that the incidence was not reported or was reported but was <10%. TEAE, treatment-emergent adverse event.

ESM Figure 1. Study design schematic.

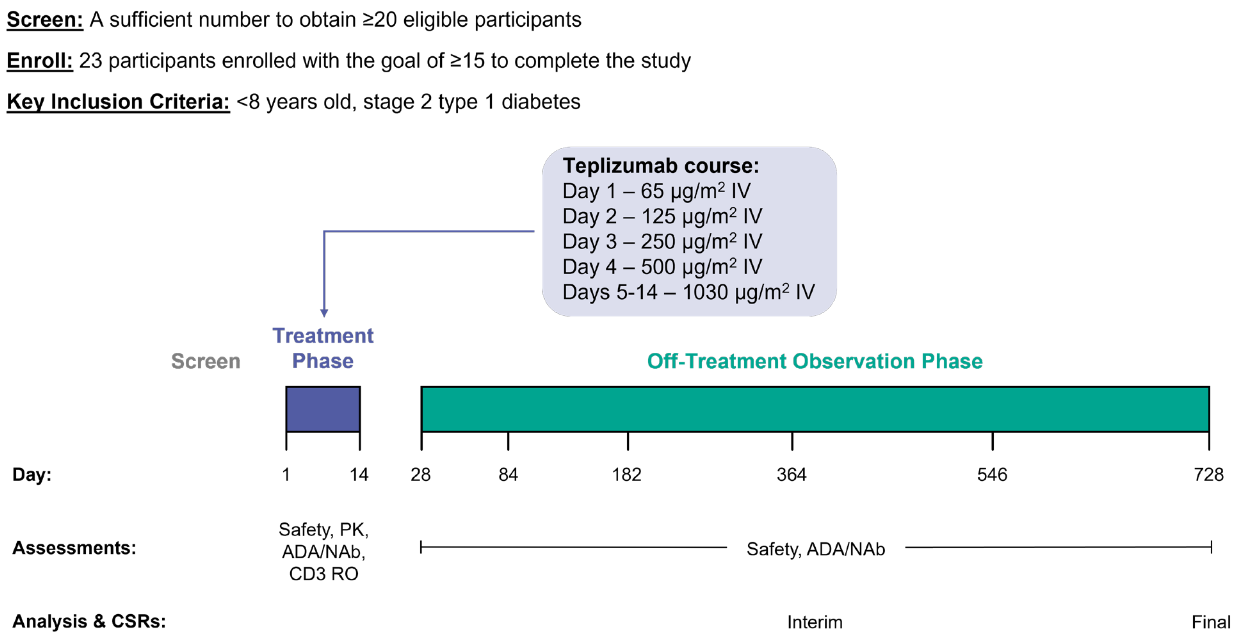

Design of the PETITE-T1D trial. First teplizumab dose occurred on Day 1 and occurred daily through Day 14. ADA, antidrug antibody; CSR, clinical study report; IV, intravenous; NAb, neutralising antibody; PK, pharmacokinetics; RO, receptor occupancy.

ESM Figure 2. Clinical trial flowchart.

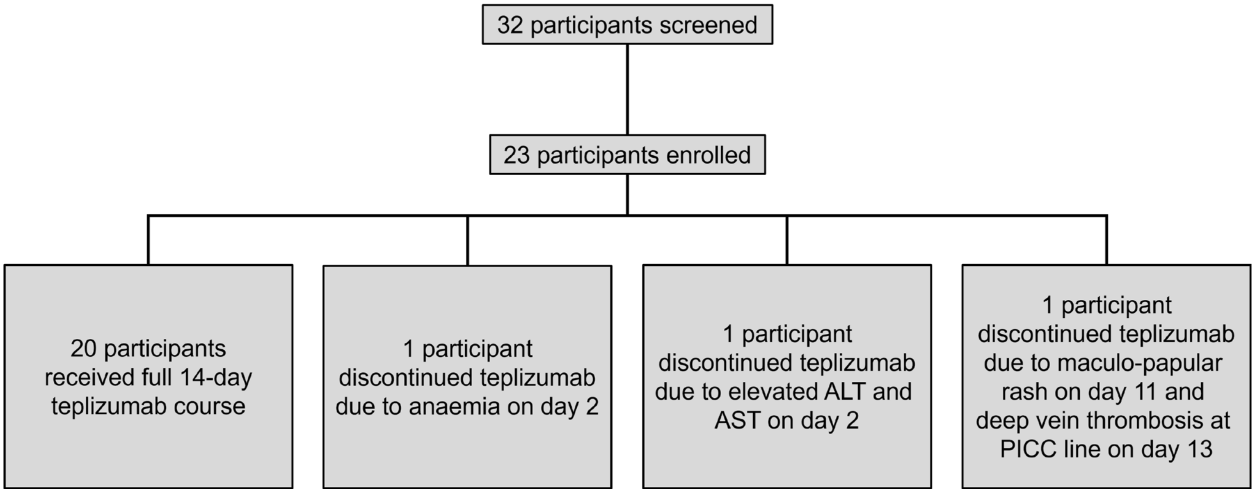

ALT, alanine aminotransferase; AST, aspartate aminotransferase; PICC, peripherally inserted central catheter.
